# Technical Advances and Pitfalls in Head and Neck Radiotherapy

**DOI:** 10.1155/2012/597467

**Published:** 2012-05-30

**Authors:** Upendra Parvathaneni, George E. Laramore, Jay J. Liao

**Affiliations:** Department of Radiation Oncology, University of Washington, Seattle, WA 98195, USA

## Abstract

Intensity Modulated Radiotherapy (IMRT) is the standard of care in the treatment of head and neck squamous cell carcinomas (HNSCC) based on level 1 evidence. Technical advances in radiotherapy have revolutionized the treatment of HNSCC, with the most tangible gain being a reduction in long term morbidity. However, these benefits come with a serious and sobering price. Today, there is a greater chance of missing the target/tumor due to uncertainties in target volume definition by the clinician that is demanded by the highly conformal planning process involved with IMRT. Unless this is urgently addressed, our patients would be better served with the historically practiced non conformal radiotherapy, than IMRT which promises lesser morbidity. Image guided radiotherapy (IGRT) ensures the level of set up accuracy warranted to deliver a highly conformal treatment plan and should be utilized with IMRT, where feasible. Proton therapy has a theoretical physical advantage over photon therapy due to a lack of “exit dose”. However, clinical data supporting the routine use of this technology for HNSCC are currently sparse. The purpose of this article is to review the literature, discuss the salient issues and make recommendations that address the gaps in knowledge.

## 1. Introduction

Radiotherapy with or without chemotherapy is the only curative nonsurgical treatment for head and neck squamous cell carcinomas (HNSCCs) and achieves high rates of local tumor control of over 80% for stage 1-2 and 60–70% for stage 3-4 [1]. Technical advances in radiotherapy during the last decade and a half have revolutionized the treatment of HNSCC. The most tangible clinical gain has been a distinct reduction in long-term morbidity in these patients. This, in large part, is almost a direct consequence of highly conformal intensity modulated radiation treatment (IMRT) planning designed to treat tumors and regions at risk for microscopic spread while sparing uninvolved normal structures, in particular, the salivary glands. Randomized controlled trials [1–3] demonstrating a superior morbidity profile in patients treated by IMRT compared to historical/conventional radiation treatment (HRT) techniques have established IMRT as the current standard of care in the treatment of HNSCC. However, there are several caveats involving the safe utilization of IMRT including target volume definition by the clinician which will be a subject of discussion in this paper. On-board daily imaging, for example, with cone beam CT is another noteworthy advance that has made image-guided radiotherapy (IGRT) practical and ensures the level of accuracy warranted to deliver a highly conformal treatment plan. The benefits and limitations of IGRT are addressed. During a course of radiotherapy, targets as well as normal structures may undergo deformations, and adapting to these changes with replanning is being investigated by several centers. Proton therapy is becoming increasingly available as a number of centers have been built in the last few years and several more are being planned or under construction. There are theoretical physical advantages of protons over photons, although clinical data are sparse and the cost is significant. The indications for proton therapy in the treatment of HNSCC are currently not well defined, and the available data is discussed.

## 2. Intensity-Modulated Radiation Therapy (IMRT)

**Figure 1:**
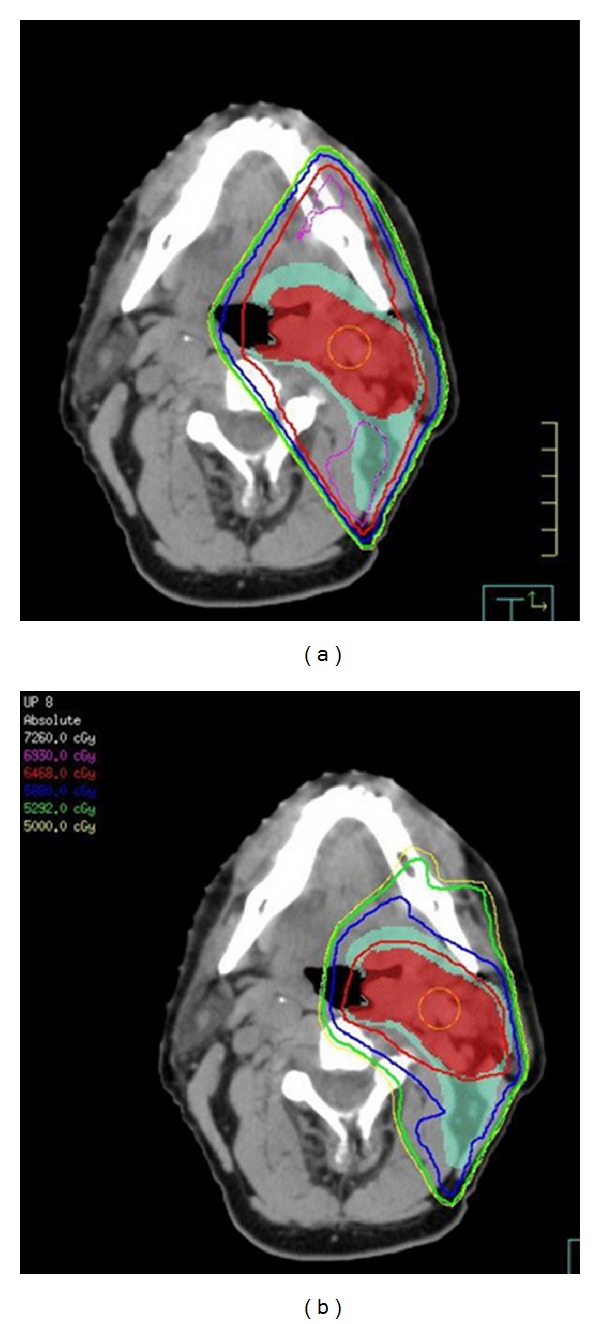
Comparison of a HRT plan (a) with an IMRT plan (b). Red color wash is the high-risk clinical target volume (CTV 66). Green color wash is the low-risk target volume (CTV 54) for microscopic disease. CTV66 is covered conformally by the red 98% isodose line in the IMRT plan. In the HRT plan conformality is lacking as the 98% isodose line covers not only CTV66 but also CTV54 as well as normal tissue including the mandible and oral mucosa. The magenta isodose line is an undesirable 105% “hot spot” located on the mandible and in the posterior neck in conventional plan. There are no “hot spots” in the IMRT plan.

IMRT is a conformal 3-dimensional technique capable of precisely targeting tumors while avoiding normal structures and is the current standard of care in the treatment of HNSCC. IMRT refers to a controlled modulation of intensity across each individual beam so that the desired high-dose distribution matches the tumor/target in all physical dimensions. Advances in computerized treatment planning systems utilizing complex inverse planning algorithms, along with hardware improvisations including high-resolution multi-leaf collimators, have made this technique readily available for routine use at most centers in the developed world. The major benefit of IMRT compared to HRT is the sparing of critical structures while conforming the desired high doses to the tumors; an example is seen in Figure 1.

**Figure 2:**
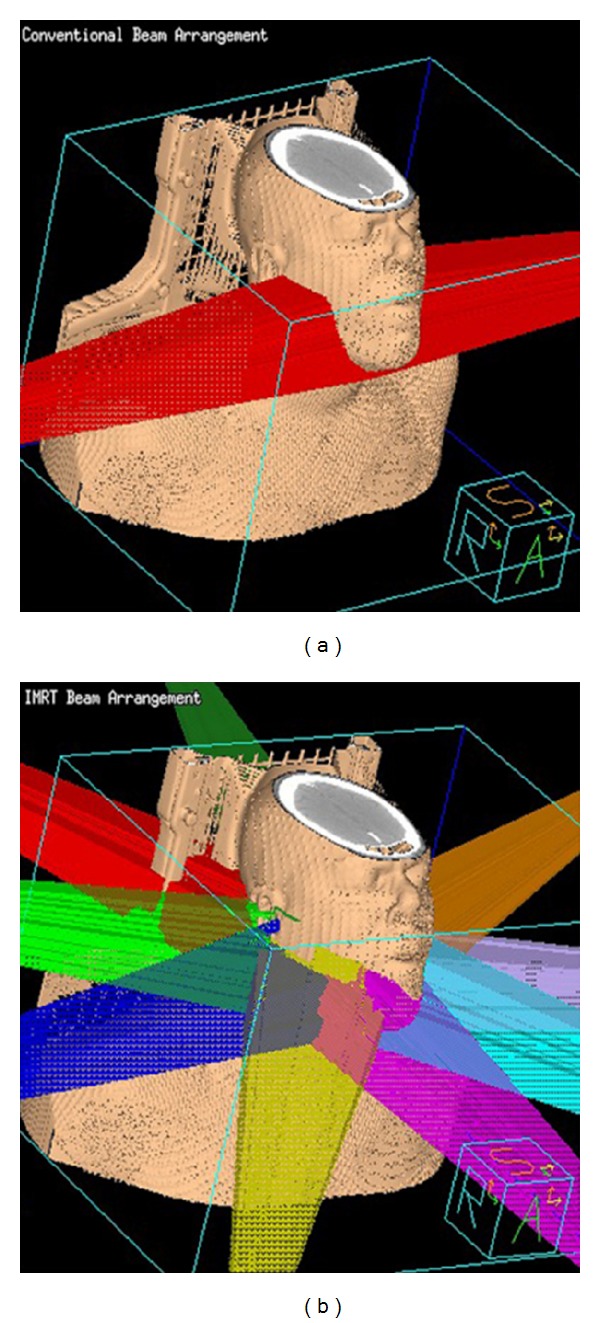
Typical beam arrangement applied for treating HNSCC of oropharynx with HRT on left using opposed lateral beams treating the primary site and upper cervical nodal regions compared with multiple beam angle arrangements employed for IMRT on right. The opposed beams do not treat through the oral cavity or the posterior scalp, and hence mucositis, is not seen in the lips and anterior oral cavity. With IMRT mucositis may be seen in the lips as well as hair loss which is observed in the posterior scalp.

The most prevalent and a highly distressing long-term complication after radiotherapy for HNSCC is xerostomia [4]. In addition to a subjective perception of a dry mouth that is unpleasant for most, lack of saliva also makes it difficult to speak, swallow, taste, chew, and wear dentures. It may contribute to nutritional deficiencies, predisposing to painful mucosal fissures and ulcerations, and adversely affects oral health, promoting dental caries, and the consequential dental extractions may contribute to osteoradionecrosis. Xerostomia is associated with significant deterioration in the patient's quality of life [4]. In selected patients IMRT can successfully spare the salivary glands mitigating these debilitating effects of xerostomia. Prospective randomized clinical trials [1–3] have demonstrated that IMRT is significantly superior to HRT in sparing parotid glands and the consequential recovery of salivary function, reducing the incidence of xerostomia with marked improvements in associated quality of life. IMRT can also spare other normal structures such as cochlea, oral mucosa, temporomandibular joint, and mandible [5, 6]. IMRT is expected to reduce the frequency of osteoradionecrosis [7] and may decrease dysphagia by reducing the doses delivered to pharyngeal constrictor muscles [8]. Although IMRT allows for better conformity of the high-dose region to the tumor, it does so at the expense of delivering low doses to a greater volume of normal tissue. This “low dose spill” into nontarget structures may result in unexpected and unintuitive short-term toxicity from IMRT including alopecia and acute mucositis in locations that were not in the beam pathways of HRT due to the multiple beam angle arrangements employed for IMRT as seen in Figure 2. Nausea from dose to brain stem (area postrema) and increased acute fatigue compared to HRT have also been reported with IMRT [1, 9]. There is a theoretical long-term risk of increased radiation-induced secondary malignancies due to the greater whole body integral dose from this “low dose spill” [10].

As with any precision technique, it is possible to be highly precise and precisely inaccurate. The greatest risk with IMRT is to miss the tumor altogether while attempting to be very precise in defining targets and sparing normal structures. The adage of “the most radio resistant cell being outside the radiation portal” holds. With historical techniques, for example, parallel opposed lateral fields matched with a supraclavicular field, the probability of missing the tumor was low, although the technique was not as precise in limiting dose to normal structures. Therefore, the success of such a highly conformal planning process as IMRT is highly sensitive to two major factors:

(1) delineation of tumor/target volume in 3 dimensions by the clinician around which the high-dose distribution is developed through conformal planning, and

(2) day-to-day variations in setup that could result in the tumor “sneaking” outside of the high-dose distribution of the conformal plan, thus missing the intended treatment dose altogether.

Several institutions have reported clinical outcome data showing excellent local control results utilizing IMRT for HNSCC [11]. In fact, the highly conformal dose distributions achieved by IMRT may improve tumor control rates in advanced cancers, in particular those arising from the nasopharynx and sinonasal regions because they facilitate the delivery of high doses to the tumor that is intimately related to adjacent critical organs like the brainstem and optic nerves, without exceeding the normal tissue tolerance. However, it is unclear whether these results generated from academic centers with a high-volume load of head and neck cases could be generalizable to community practices that see a lower volume and yet have embraced IMRT as the standard of care. In an effort to investigate whether the early successes of IMRT reported by a few institutions could be reproduced in a multi-institutional setting, the Radiation Therapy Oncology Group (RTOG) embarked on a prospective study of IMRT for early oropharyngeal cancer [12]. This study was the first multi-institutional trial incorporating IMRT with participation from 14 institutions and accrued 69 patients. It included guidelines for target definition, target dose prescription, and tissue dose constraints. The trial also used central quality assurance (QA) processes assessing the ability of the participating institutions to plan and execute IMRT and the quality of the individual IMRT plans. In this study, major target underdose deviations were associated with a significantly higher locoregional failure rate of 50% compared with 6% without such deviations. As with any technical skill, it is quite possible that a learning curve exists in the use of IMRT and that experience gained by treating a high case volume of patients might reduce the risk of failure. For example, three randomized studies [13–15] demonstrated that in a controlled clinical trial setting, larynx preservation is possible without compromising survival in advanced larynx or hypopharynx cancers that would have otherwise required a total laryngectomy. As a result, this strategy of laryngeal preservation for advanced larynx cancers was widely embraced by the oncology community. However, recent data [16, 17] highlighted a decrease in survival corresponding with a change in care pattern of advanced laryngeal cancers from total laryngectomy to conservative treatment with radiotherapy with or without chemotherapy, especially when treated in low-volume cancer centers. This could well be a consequence of poor case selection and/or technical insufficiencies by Radiation Oncologists practicing in the community and seeing a low-case volume. Other studies [18–21] have identified that in head and neck and other cancers whose treatment is predominantly driven by technical expertise, a higher case volume is a predictor of favorable outcome compared to a lower case volume.

The highly conformal dose distributions produced by modulation of the beam intensity require careful delineation of the gross tumor and high- and low-risk clinical target volumes as well as the surrounding normal structures. Inadequate delineation of the target volumes can diminish tumor control or increase toxicity with IMRT. Patterns of failure that are peculiar to parotid sparing IMRT with unsalvageable relapses at the skull base [22] and periparotid failures [23] have taught us the importance of recognizing the limits of sparing normal structures and the knowledge gaps that exist regarding microscopic tumor spread.

Contouring of target volumes can vary even among expert radiation oncologists. Cooper et al. [24] reported the discrepancies in contouring of supraglottic carcinomas based on CT images between 8 leading experts in head and neck cancer management including 4 radiation oncologists and 4 neuroradiologists. The average proportion of overlap (i.e., the degree of agreement) was approximately 50%. They concluded that “the estimation of tumor shape currently is imprecise, even for experienced physicians. In consequence, there appears to be a practical limit to the current trend of smaller fields and tighter margins that is typically employed with IMRT in an attempt to maximize sparing of normal structures.” In another study comprising of international experts involving 20 institutions in the United States, Europe, and Asia treating HNSCC, Hong et al. found remarkable heterogeneity in target delineation [25]. There was a fivefold variation in clinical target volumes for the ipsilateral neck (range, 35–175 cm^3^; mean, 120 cm^3^). Similar variation was identified for treatment volumes in the contralateral neck, if treated. Wide variation also existed in the specific nodal stations included within the designated targets. If experts disagree to the degree pointed above, one can only but imagine the discomfit experienced by an average radiation oncologist who does not see a huge volume of HNSCC but feels obliged to treat his/her patients with the current standard of care, that is, IMRT.

Consensus guidelines are available for delineating the elective targets that are the presumed pathways of spread through regional lymphatics [26, 27], but not for the high-risk regions that immediately surround the gross tumor. Besides, the infinite subtle anatomical variations between patients preclude replication of contours based on modal cases that are depicted in guidelines, and clinical judgment is required more often than not. Eisbruch et al. reported that despite clear and well-written guidelines that were formalized by many clinicians and physicists after considerable debate, overall not a single case was judged to be according to protocol without any variation in the RTOG 0022 study [12]. This speaks to the tremendous variation in the interpretation of guidelines in “real life” clinical practice. Of course, image fusion with MRI scans and PET scans could further improve the accuracy of contouring, but since no test is 100% sensitive and specific, none of these additional tests can replace the clinical judgment that is required for contouring. Adequate contouring skills call for a detailed knowledge and understanding of 3-dimensional anatomy and an ability to perform a thorough physical exam as the extent of the gross tumor cannot be reliably gleaned from imaging alone [28]. One is expected to learn the art of physical examination “on the job” through clinical rotations during residency training. However, as far as we are aware there are no specific head and neck physical exam training sessions in any of the radiation oncology training programs. In addition, there are no available resources that are instructive in “translating” physical exam findings to 3D images that radiation oncologists rely on to contour tumor volumes. Further discussion of this topic is beyond the scope of this paper.

It is encouraging to note that contouring target volumes is a skill that can be taught. Bekelman et al. [29] reported on the results of a teaching intervention on a group of 11 radiation oncology residents who were primed with 2 seminars (6 × 1 hr per seminar) on the principles of head and neck radiation oncology. They then underwent a baseline contouring evaluation on a mock case. This was followed by a didactic lecture and an interactive practical session. Follow-up evaluation took place after 8–10 days on the same baseline case. Improvements in contouring skills were noted in all the participants for all the target volumes and 100% adequately contoured the high-risk target volume following the teaching intervention while only 60% of those never completed a head and neck clinical rotation and 83% of those who completed one could do so at baseline, although all have been primed with 2 seminars. While the study was limited in that it was a small sample from a highly select group of trainees, and the gross tumors were precontoured, it highlights important points. Despite the priming seminars and over half the participants having gone through clinical rotations, many failed the baseline evaluation. Second, an effective and targeted teaching intervention could impart this essential skill.

We suggest the following for radiation oncologists treating HNSCCs with IMRT.

(1) Radiation oncologists practicing in low-volume centers are to maintain their skills periodically (e.g., annually) by attending an intensive and targeted teaching course, for example, ESTRO educational courses, Princess Margaret Hospital's IMRT course, and so forth. Currently, groups that offer such courses do not have a stringent/standardized evaluation component to examine the learner and provide individualized feedback for further learning and development. It would also be advisable to establish a mentorship relationship with a leading expert from the teaching course to discuss complex cases and, if possible, get a virtual QA of contours for all their IMRT cases until they achieve a level of concordance with the expert who is mentoring them. This is in keeping with the broad principle of continuing medical education.

(2) For those practicing in high-volume centers, a target volume QA program that verifies individual contours is highly desirable. For example, at the University of Washington (UW) every patient's target volume contours are cross-checked by an independent expert other than the treating clinician. We also incorporate critical aspects of physical examination during these sessions utilizing clinical photographs and nasoendoscopy videos that are recorded at the time of simulation. Where logistically feasible, the independent expert also examines the patient together with the treating clinician. Consensus target volumes are then developed for IMRT planning.

(3) While peculiar patterns of failure have been identified at academic centers [22, 23], the failures in the community setting are largely unknown. It behooves every radiation oncologist who is utilizing IMRT to carefully follow their patients and analyze and report any failures. This is an extremely important step for the continued development of this novel and exciting technology.

## 3. Image-Guided Radiation Therapy (IGRT)

An integral component of the treatment process that is essential for the success of IMRT is the accuracy of patient setup. Due to the steep dose gradients that are achievable with IMRT, the margin for setup error is small and the accuracy of daily alignment and positioning becomes even more important as there is an increased risk for a “marginal miss” and underdosing of the tumor as well as unintended high doses to organs at risk.

The most basic and historical form of IGRT consisted of two-dimensional (2D) portal images acquired in perpendicular/orthogonal planes to verify the position of the isocenter, as well as the individual fields. In most cases, these images are generated by the megavoltage (MV) beam of the treatment machine or less frequently by a dedicated ancillary kilovoltage (KV) unit. Bony anatomical landmarks of the spine and skull are typically used as reference for alignment. These images are obtained prior to treatment at our institution on the first 3 days of treatment and thereafter once a week, but other schedules have also been used. The limitations are that bony image quality is often quite vague especially with MV imaging, and small displacements may not be readily or reproducibly identified, and clinical judgment is often required to interpret these images. Historically, it is the therapists/technicians with a limited knowledge of the cancer and the planning process that are making these qualitative decisions regarding the accuracy of setup. In addition, soft tissue alignment and deformations cannot be appreciated with 2D imaging.

Recent advances in 3-dimensional or volumetric imaging have addressed some of these issues, and cone beam CT (CBCT) has emerged as an efficient system for in-room localization. Essentially, this consists of a compact CT scanner that is integrated into the linear accelerator unit. A scan can be acquired quickly, generally in 1 to 2 minutes, just prior to treatment with the patient in the treatment position. This scan is generally of lower resolution than a diagnostic CT scan but provides sufficient bony and soft tissue resolution for anatomic alignment. Both KV and MV CBCT systems are available, but KV imaging provides better image contrast and signal-noise ratio and is used at our center. The CBCT localization scan is then superimposed on the treatment planning CT scan utilizing a software-based registration algorithm to verify the accuracy of setup, and any necessary shifts are made to obtain an accurate match. In order to circumvent setup errors IGRT that verifies the “match” between the treatment plan and final patient position and corrects for inaccuracies is currently being adapted at several centers.

**Figure 3:**
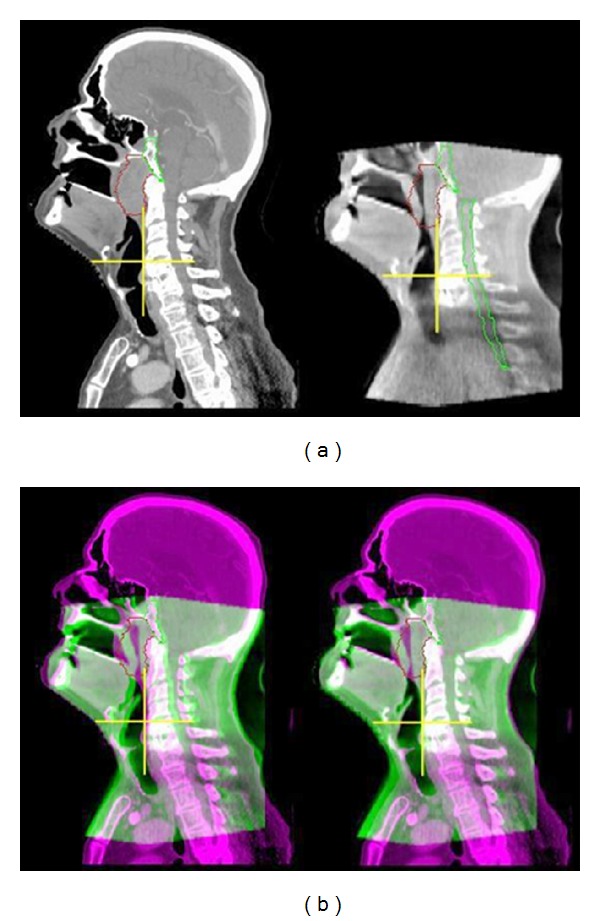
(a): Patient with locally advanced nasopharyngeal cancer showing CT simulation planning CT and a corresponding daily CBCT localization scan acquired during week 4 of treatment. Primary tumor is outlined in red. Note the interval tumor regression evident on CBCT. (b): Superimposed planning CT and CBCT demonstrating obvious anterior-posterior misalignment before then after image-guided correction.

Treatment planning target volumes, organs at risk, and/or any user-defined regions of interest (ROI) or structures may be visualized. Bony misalignments are readily identified, rectified, and the offsets are automatically recorded. In addition, soft tissue deformations of tumor (e.g., due to regression), as well as normal structures (e.g., due to weight loss) may be identified, and it raises the possibility of “adaptive radiotherapy” (ART) in response to these changes (Figure 3).

Immobilization is a vital component of treatment to decrease setup errors and facilitate safe delivery of treatment. Standard immobilization for planning and treatment of HNSCC is achieved with a thermoplastic mask, and it is generally believed that this setup is stable. However, recent studies [30, 31] have reported considerable setup variations with a standard mask immobilization. Ahn et al. performed repeat simulation planning scans 3 times in a series of patients with HNSCC during their treatment course [30]. They observed significant random positional variation in the bony anatomy, that is skull, mandible and cervical spine despite mask immobilization, with the effects most pronounced for the mandible and lower cervical spine. Hong et al. reported a series of patients treated with daily localization using an optical guidance system comprised of an infrared radiocamera and passive fiducial arrays [31]. Accounting for all 6 degrees of freedom, the mean vector offset or setup error was 6.97 mm (SD 3.63). They reviewed the dosimetric impact of this on theoretical IMRT plans and found that geographic “tumor miss” and normal tissue overdosing was common. Taken together, these studies call for an improvement in the immobilization system and/or routinely perform IGRT to verify patient positioning and ensure the required accuracy to safely treat with an IMRT plan. Various strategies for thermoplastic mask external immobilization may impact on setup accuracy [32]. Internal immobilization, where feasible should be optimized. Customized oral stents may be used for reproducible immobilization of the tongue in addition to their role in maximizing normal mucosal sparing [33].

Despite mask immobilization, the flexibility of the neck and shoulders could also lead to deformations with setup errors even with IGRT. Differential planning target volume (PTV) margins might be necessary for different regions of the neck, especially with a whole neck IMRT technique. Image registration is generally performed at the level of the primary tumor using a bony match. However, there could be anatomic displacements within the treatment field at a location that is further away from the primary site. For example, for tonsil cancer the match is obtained at C2-3 vertebral bodies, but there could be a mismatch in the low neck below the C6 level that is necessary to electively treat the level 4 lymph nodes.

Ahn et al. demonstrated that there can be random variation and semi-independent rotational and translational movement of the skull in relation to the lower cervical spine [30]. Polat et al. evaluated nonrigid setup errors in a series of patients treated with daily CBCT with different regions of interest in the head and neck used for automatic registration [34]. They found that the greatest mobility was in the skull and mandible relative to C4–C6. Necessary margins for compensation of this relative motion ranged between 5 and 10 mm, which exceeded typical PTV margins. Ove et al. reviewed a series of patients undergoing IMRT with daily image guidance using a CT-on-rails platform with planning margins of 2–5 mm [35]. Patients were generally matched to high neck anatomy at C1-C2. They found that a low neck point (defined anatomically) relative to this coordinate was displaced by an average of 3.08 mm anteriorly (±0.17 mm). There were significant random setup errors in the low neck with standard deviations of 3.9 mm, 3.3 mm, and 2.6 mm for anterior/posterior (AP), medial/lateral (ML), and craniocaudal (CC) displacements.

Den et al. recently reported one of the largest prospective series to date with daily pretreatment CBCT-based IGRT in 28 patients undergoing head and neck IMRT [36]. Base of skull lesions (nasopharynx and sinonasal) was aligned using bony match and others (oral cavity, oropharynx, and larynx) with grey value match. At least once a week, a post-treatment CBCT was obtained for additional QA. CBCT typically increased patient time on the table by no more than 3 minutes, and this additional time on the treatment table was tolerated well by the patients. The average shift for pretreatment CBCT scans was 1.40, 1.66, and 1.79 mm for the ML, CC, and AP directions. Shifts of ≥3 mm were required in 11% of setups in the ML, 14% in the CC, and 17% in the AP direction. In this study, the necessary margin expansions from clinical target volume (CTV) to PTV in order to account for daily setup variation were found to be 3.9, 4.1, and 4.9 mm in the ML, CC, and AP dimensions, respectively, without CBCT to account for systematic and random errors. Therefore, they recommended a minimum of 5 mm CTV to PTV margin expansion in HNSCC patients treated without CBCT. With daily CBCT, they proposed that margins could potentially be reduced to 2-3 mm. Preliminary clinical data [37] based on 130 patients with HNSCC treated with IMRT using a 3 mm CTV to PTV expansion suggest this is safe as they were compared with an earlier cohort treated using a 5 mm expansion. IGRT was achieved using KV cone beam or MV fan beam using automated registration bone presets. There was no difference in two-year estimates of overall survival and locoregional control, and there was no difference in the incidence of marginal failures. Further follow-up and additional studies will be required to confirm the safety of margin reduction with IGRT.

A reduction in margin, if performed safely, could allow for greater sparing of normal structures. Van Asselen et al. evaluated the impact of margin reduction on parotid preservation with IMRT for oropharyngeal cancers and found that a PTV margin reduction from 6 mm to 3 mm resulted in a 20% reduction in normal tissue complication probability or about 1.3 Gy mean parotid dose per 1 mm [38]. It may also open the door for dose escalation. However, extreme caution is necessary with these highly conformal approaches given the close association of dose and locoregional control in HNSCC. PTV margin reduction below 5 mm in the absence of daily IGRT is probably inadequate [36, 37, 39]. Even with IGRT, Den et al. [36] caution that tumors arising from mobile structures (e.g., larynx, tongue) and patients who lost weight during treatment required a greater margin.

In a randomized study [1] there were greater numbers of failures in the IMRT arm compared to HRT arm (12 versus 7) within the “high dose” field. This study was not powered to detect locoregional failures, and this finding was not statistically significant but is concerning all the same. This study had target contouring guidelines, but the expansion from CTV to PTV to account for day-to-day variations in setup was only 3 mm [40]. This study was conducted in the era before the advent of modern IGRT techniques. Although this is tantalizing data, one could reasonably speculate that the 3 mm expansion was inadequate in the absence of rigorous daily IGRT while utilizing highly conformal IMRT plans to treat patients. The failures that were coded as “within the high dose volume” based on the initial IMRT plan could have actually been marginal due to the target being marginally missed by day to day “minor” setup variations.

However, there are several other unresolved issues with IGRT.

Varying approaches have been used with regard to the frequency of imaging. Zeidan et al. evaluated several IGRT protocols in patients with HNSCC with varying percentage of fractions ranging from 0% to 50% [41]. The protocols included no imaging, initial fraction only, initial 3 versus 5 versus 7 fractions followed by mean shift and thereafter weekly imaging with a 3 mm threshold, or imaging every other fraction with a running mean. Image registration was performed using a bony anatomy-based automatic fusion algorithm. Random setup errors were not reduced for fractions without image guidance. Protocols requiring 15–31% of treatments to be image guided were subject to setup errors >5 mm in 26–31% of fractions and >3 mm in 50–60% of fractions. Every other day image guidance was associated with setup errors >5 mm in 11% of fractions and >3 mm in 29% of fractions. They concluded that systematic setup errors were reduced with increasing frequency of image-guidance, but not random errors. Our institutional policy for IGRT specifies daily imaging to address both systematic and random setup errors. The clinical relevance of cumulative dose from IGRT is unclear.

The radiation dose from a single CBCT scan is almost negligible, but the cumulative dose from frequent CBCT imaging over a course of 30–35 treatments may not be so. It is possible that this additional low dose to normal tissues might increase the risk of radiation-induced secondary malignancy, in the same way as the theoretical risk due to the greater whole body integral dose from the “low dose spill” from IMRT [10]. This may be of particular concern in pediatric patients. Currently, there is no consensus in whether this “imaging dose” should be factored into dose calculations during treatment planning.

The optimal approach for image acquisition for IGRT has not been defined and would depend on the specific CBCT platform used and the needs of the individual patient. There are a number of parameters that may impact on image quality including the imaging field of view (FOV), rotation arc and acquisition time, and use of imaging filters [42]. The method used for image registration and evaluation can have a large impact on the resulting shifts and setup accuracy. Some of the major factors to consider are whether registration is performed over the entire image or a specific ROI defined by a clip box, automatic versus manual matching, soft tissue/grey-scale versus bone versus multiple ROI matching, degrees of freedom available for positional correction (i.e., translational only versus translational and rotational), strategy for postcorrection verification, online and/or offline corrections, and frequency of imaging. The ability and training of physicians and therapists to verify image registration and how image guidance is integrated into a specific clinic's workflow are also important considerations. Familiarity with these issues is essential before initiating routine clinical application.

At our institution, site-specific CBCT protocols for image registration and setup correction for HNSCC have been defined. Based on broad general principles, we also developed guidelines for the clinical indications of IGRT with CBCT and some examples are discussed below.

**IMRT Plans with Steep Dose Gradients**

**Figure 4:**
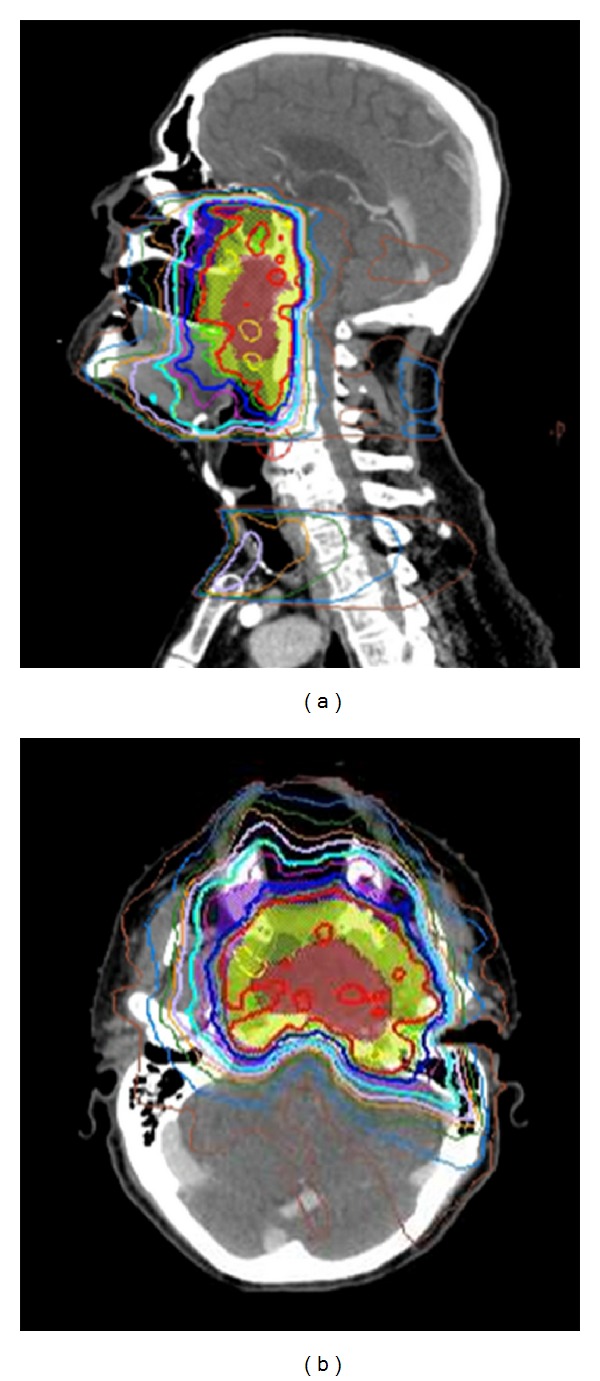
IMRT plan for the same patient above with a locally advanced nasopharyngeal cancer demonstrating sharp dose gradient at the level of the skull base, clivus, and upper cervical spine in close proximity to temporal lobe brain, brainstem, and spinal cord. CBCT was used for daily alignment to ensure setup accuracy (see Figure 3).

IMRT plans with target volumes in close proximity to critical structures including nasopharyngeal, nasal cavity and paranasal sinus, orbital and periorbital tumors where PTVs are in close proximity to brainstem, spinal cord, temporal lobes, and optic apparatus (eyes, optic chiasm, optic nerves). Daily CBCT ensures the accuracy of setup necessary to deliver these plans (Figure 4).

**Bulky Exophytic Tumors**

Bulky exophytic tumors often experience significant tumor regression during the course of treatment (Figure 3) and may benefit from daily CBCT to help guide the timing of ART and limit mucositis. Conversely, it may be feasible to identify resistant tumors that do not regress as anticipated and boost them to a higher dose.

**Infrahyoid Primary Tumors**

Larynx, hypopharynx, or thyroid cancer treatment with IMRT may benefit from daily CBCT to ensure accurate alignment with respect to the mid/lower cervical and upper thoracic spine. Set-ups in the lower neck below the level of the hyoid are inherently less stable due to positional variation of the neck, and bony anatomy is often difficult to visualize in the low neck near the clavicles on traditional port films.

**Reirradiation**

It is imperative to verify accurate setup given the elevated risk of normal tissue complications, for instance, tight restrictions with spinal cord tolerance dose due to prior treatment may benefit from daily CBCT.

**Hypofractionated Regimens**

Patients undergoing treatment with hypofractionated regimens such as high-dose palliation (dose per fraction >/= 3 Gy and total dose >/= 45 Gy) may benefit from CBCT to verify treatment accuracy given the limited number of high-dose fractions and the risk of exceeding critical structure tolerance with hypofractionation.

**Other**

There are special situations where there is concern of unstable setup and/or difficult verification by traditional portal images, for instance, a patient with severely osteopenic bones or degenerative changes that could render portal images difficult to interpret.

## 4. Additional Topics Pertaining to IMRT

### 4.1. Adaptive Radiotherapy (ART)

ART is a process of adjusting the treatment plan in response to changes observed during radiation treatment. Deformations of targets, normal structures as well as patient anatomy may occur during a 6-7 week course of radiotherapy. For example, Bulky exophytic tumors, in particular oropharyngeal tumors of human papilloma virus origin and nasopharyngeal tumors in the Asian population, often experience significant tumor regression during the course of treatment (Figure 3). It may be feasible to replan to adjust for interval regression of the exophytic component of the disease to limit oral mucositis. Conversely, it may be feasible to identify resistant tumors that do not regress as anticipated and boost them to a higher dose. These scenarios are currently being investigated by several groups to mitigate the problem of target deformation during a treatment course. Apart from physical deformations in targets, there could also be biological variations with redistribution of tumor cells through phases of cell cycle and reoxygenation of previously hypoxic cells converting radio resistant cells to radiosensitive in some cases and vice versa in others. Currently, there is no test that can provide unambiguous information about these changes. Other changes in patient anatomy from weight loss and tissue edema may also occur during treatment. All these could have an impact on the dosimetric parameters and potentially translate to a clinically significant impact both on tumor coverage and normal tissue toxicity. For instance, it has been observed during the course of radiotherapy that the parotid glands may migrate medially during a course of radiotherapy, which may impact on the expected versus actual doses to these structures [43]. IGRT with CBCT provides important anatomic information during the course of treatment, which could be the ideal platform to facilitate ART. Currently, soft tissue resolution is quite limited compared to bony anatomy. Although a number of groups have reported early experience with ART for HNSCC [44–47], the optimal strategy, frequency, and clinical impact are not well established, and at this point in time ART is considered explorative.

### 4.2. Volumetric Modulated Arc Therapy (VMAT)

The toxicity associated with treatment of HNSCC, for example, painful mucosal reactions, thickened secretions that are difficult to clear, dysphagia, exaggerated gagging, along with a sensation of claustrophobic anxiety while lying under the mask contributes to setup instability on the table and could be associated with unrecognized intrafraction motion. These aspects can vary from patient to patient and should be considered in the design of PTV margins. There is an increase in treatment time per fraction from approximately 5–10 minutes for a conventional treatment to 20–30 minutes with IMRT. The longer a patient is on the treatment table, the more likely he/she will move and hence the unreliability of positioning during setup and treatment. Emerging radiation techniques such as VMAT are capable of generating conformal plans that are comparable to IMRT but use shorter treatment times that are comparable with HRT [48].

## 5. Charged Particle Radiotherapy (CPR)

**Figure 5:**
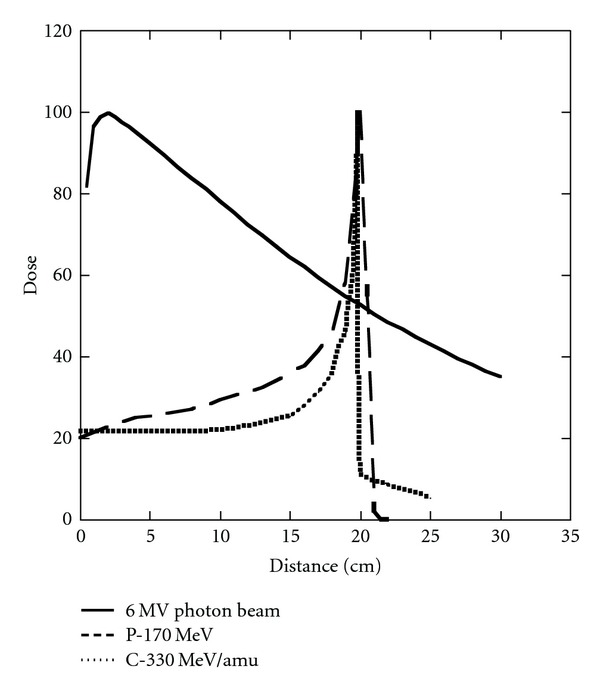
Depth dose curves for a 6 MV photon beam (solid line), a 170 MeV proton beam (dashed line), and a 330 MeV/amu carbon ion beam (dotted line) as a function of depth in a water phantom. The beams have been arbitrarily normalized to 100 at their maxima.

Unlike photon beams, charged particle beams have sharp cutoffs in their range due to the intrinsic physical principles underlying their interactions with matter. They deposit little energy until they near the end of their range at which point the rate of energy loss increases resulting in what is termed a Bragg peak.  Figure 5 shows the energy loss in water for a typical megavoltage X-ray beam used in therapy, a proton beam, and a carbon ion beam with energies set to place the Bragg peaks at about a 20 cm depth in water. For practical clinical purposes, protons have the same biological properties as photon beams, apart from a small scaling factor of 1.1, which is taken to be the same for all tissues [49]. This ignores a very small region at the distal edge of the Bragg peak where increased linear energy transfer (LET) theoretically should result in an increase in relative biological effectiveness (RBE). For heavier ions such as carbon, the high LET along their path gives rise to higher RBEs, similar to fast neutrons, which are both tissue and dose regimen dependent. While the lateral beam edge is sharper for C-ions than for protons, fragmentation effects give rise to a “tail” at the distal edge of its Bragg peak.

CPR centers utilize cyclotrons or synchrotrons to accelerate the particles to hundreds of MeV/amu and then direct the beam to one of several treatment rooms. Initially, the position of the beams was fixed in the room, generally in the horizontal direction, but more recently, rotating gantries are used which allow the treatment of tumors located anywhere in the body. When the particle beam emerges from the accelerator, it has a cross-section of only a few millimeters. Special techniques are required to shape the beam to the target volume. The initial approach was to use scattering devices to spread the beam both horizontally and along the beam axis [50]. Collimators and energy absorbing compensators further shape the beam. More recently, scanning magnets controlled by computers, coupled with beam energy control systems, allow the scanning of the Bragg peak throughout the target volume and the delivery of intensity modulated proton therapy (IMPT) [51–53]. This produces fewer extraneous neutrons in the treatment room compared to the scattering technique, something of potential importance in reducing the risk of second malignant tumors [54].

HNSCCs are good test systems for CPR because of the complex treatment volumes and close proximity of high-risk regions to critical avoidance organs at risk such as spinal cord, brain stem, and optic chiasm as well as to less critical avoidance organs such as salivary glands, hearing apparatus, and carotid arteries. IMRT may produce high-dose volumes that conform well to the PTV but does so at the cost of irradiating larger volumes of normal tissues to low-intermediate doses [10]. In most cases, the “cost” of irradiating this extra tissue is not well defined, but as per the ALARA (as low as reasonably achievable) principle it would be desirable to reduce or eliminate radiation dose to normal tissue. There are two treatment paradigms that CPR can exploit in improving outcomes compared to those of photon radiotherapy: (i) strive for improvements in local/regional tumor control (which may translate into improved survival) by increasing the dose delivered to high-risk volumes while keeping the dose to normal tissues the same or (ii) keep the dose to the high-risk volumes the same with a dose reduction to normal tissues (which may translate into reduced treatment-related morbidity). Of course, hybrid approaches of the two may also be utilized.

While there have been multiple treatment planning studies [55–59] showing the dose superiority of CPR to that of photon radiotherapy, there have not been many clinical reports on its utility for HNSCC. In general, it is difficult to lower the Dmax to normal structures (OARs) when they were in close proximity to the high-dose GTV. It was in the doses to OARs located some distance away from the GTV where the CPR plans showed a major advantage over photon radiotherapy. In addition, the dose to the skin at the entry portal could be higher than would be expected with IMRT. Parvathaneni et al. [55] studied 10 cases of early stage tonsil cancers treated with unilateral technique and compared IMRT plans with Proton therapy and found that the maximum dose to skin with proton plans was 66 Gy versus 58 Gy with IMRT. The volume of skin receiving 60 Gy and above with protons was 11 cc versus 0.5 cc with IMRT. The proton plans were performed with passive double scattering and most of the plans utilized 2-3 beam angles. While these skin doses are within generally tolerable limits, it may not always be the case for larger portals or in complex reirradiation settings. A systematic review of the comparative treatment planning literature identifying studies focusing on a wide variety of HNSCC types, paranasal sinus, nasopharynx, oropharynx, larynx, and hypopharynx [59], showed that IMPT allows for dose escalation to the primary tumor without exceeding dose limits to OARs. While being important contributions to the literature, we remind the reader that simply demonstrating superior dose distributions in treatment planning comparisons does not substitute for clinical trial data in assessing the relative merits of CPR versus photon radiotherapy. In particular, CPR is often more sensitive to daily setup variation, changes in patient anatomy during treatment, and lack of knowledge of whether clinically normal treatment volumes are truly at risk for microscopic tumor spread. Moreover, the range of ion beams can be impacted by metal artifacts such as dental prostheses and surgical reconstruction plates that are often found in the head and neck cancer patient, something of lesser concern in photon radiotherapy. For example, dental fillings produce an underestimation of the ion range in the order of 3%. Tungsten prostheses are often used for postsurgical reconstruction causing streak artifacts in the CT images which result in errors of the order of 1% in the calculated path length for beams passing through them.

There are a small number of articles reporting clinical outcome data for HNSCC patients treated with CPR. Admittedly these studies are often not well controlled, and many patients were treated with a combination of photons and protons rather than with protons alone. This, coupled with the small patient numbers, makes it difficult to draw meaningful conclusions about the efficacy of the CPR component. Lin et al. describe a series of 16 patients with recurrent tumors reirradiated with protons to doses of 59.4–70.2 CGY [60]. Overall survival and local/regional control at 2 years were both 50%. No CNS toxicity was noted, but sometimes tumor coverage was compromised to ensure this. There was a notable difference in overall survival at 2 years between patients treated with “optimal” tumor coverage (83%) versus “suboptimal” tumor coverage (17%). Chan et al. discuss 91 patients with advanced paranasal sinus tumors who received combined photon and proton radiotherapy at the Harvard Cyclotron-Massachusetts General Hospital proton treatment center [61]. Ninety-one patients with AJCC stages III and IV tumors were treated during the period 1988–2002. The median prescribed dose was 73.6 CGE with 49% of the dose, on the average, being given with protons. As a function of histology, the 3-year local control rate was 83% for squamous cell tumors, 91% for carcinomas having neuroendocrine features, 86% for adenoid cystic carcinomas, and 88% for sarcomas. The overall group of patients had a 5-year survival rate of 58% with distant failure being the predominant pattern for relapse. A subsequent analysis of late visual toxicity for patients with advanced sino nasal tumors showed that the 5-year probability of having significant complications approached 20% when the median tumor dose was 70 CGE, and an accelerated fractionation schema of photons and protons was utilized [62]. Because of the heterogeneity of treatment, toxicity cannot be attributed to either component of treatment, and one cannot judge what would have happened if the patients would have been treated with protons alone.

In 1991 Loma Linda University Medical Center opened a prospective protocol using a combination of protons and photons in an accelerated fractionation schema to treat 29 patients with locally advanced carcinomas of the oropharynx [63]. An accelerated hyperfractionation technique similar to the concomitant boost approach of M.D. Anderson was utilized. A total dose of 75.9 CGY was delivered in an overall time of 28 treatment days, but only 25.5 CGY of the total dose was given with protons. No patient received concurrent chemotherapy. Twenty-nine patients were treated over 10 years. Two-year local-regional control was 93%, and disease-free survival was 81% with 16% of the patients having RTOG late toxicity scores of 3. The prolonged period of time that this study was carried out along with only about 35% of the dose being given with protons makes it difficult to ascertain the role of proton radiotherapy.

The possible increased risk of second malignant tumor induction with protons has been alluded to earlier in this section, and it is certainly something of considerable interest to those centers treating with CPR. The risk varies considerably with the region of the body being treated. Yoon et al. investigated the competing effects of higher integral dose volume for photons versus the secondary neutron bath that is produced by proton therapy [64]. Detailed comparisons were made between IMRT and proton radiotherapy for selected patients who were to receive proton RT at the Proton Therapy Center in Goyang, Korea for head and neck tumors, prostate cancer, and brain tumors. Neutron measurements made using anthropomorphic phantoms for a proton beam generated using the double-scattered passive technique with a CR-39 detector were to measure the secondary neutron dose. For IMRT treatments the secondary photon dose was assessed by measuring ionizations as function from distance from isocenter. Secondary cancer risk was estimated for stomach, lung, and thyroid using a dose-response weighted variable OED (organ equivalent dose). They found for head and neck treatments there was a lower relative cancer risk for all organs, even for the thyroid gland. Larger differences were found for the other tumor sites.

Data are even sparser for carbon ions. Mizoe et al. have reported on a phase I/II dose escalation study for patients with advanced head and neck tumors treated with carbon ions at HIMAC facility in Chiba, Japan [65]. A mixed group of tumors was treated with the overall 5-year local control rate being 75% for 34 analyzable patients. The authors concluded that the treatment toxicity was acceptable and comparable to what would have been expected with photon radiotherapy. There was improved clinical outcome for patients with nonsquamous cell tumors such as melanomas and salivary gland tumors; the same subset of tumors where another form of high LET radiotherapy, fast neutrons, showed improved results compared to standard photon radiotherapy.

Overall treatment cost is another factor to be considered when evaluating particle radiotherapy for head and neck cancer. Peeters et al. developed a model for evaluating integral costs, capital and operational costs, and relative cost per fraction for protons and C-ions compared to photon radiotherapy [66]. They attempted to identify the factors affecting cost per fraction and then applied the model to different tumor sites, one of which was head and neck cancer. The assumption was made that the particle radiotherapy centers were fully utilized; however patients were assumed to have “special category tumors” of greater complexity while the photon centers were assumed to have a mix of standard and complicated cases matching a typical utilization spectrum. Furthermore, they assumed a 30-year life cycle for equipment. This is appropriate for the particle equipment but not for the linear accelerators used in a conventional treatment center. The later would have a typical life expectancy of 7–10 years in most business model scenarios. One of key items in assessing a cost is the postulated treatment time per fraction which was taken as 30 minutes for head and neck particle treatments compared to a photon IMRT time taken of 15 minutes. The high LET of carbon ions allows for a shorter treatment course, and so the number of fractions was 16 for C-ions compared to 32 for protons and 33 fractions for IMRT. They found overall costs per course for head and neck cancer to be €30,080 for C-ions, €39,610 for protons at a proton-only facility, and €11,520 for IMRT. As might be expected from the greater capital cost, treating with protons at a combined C-ion—proton facility was considerably more expensive than if the treatment was carried out at a dedicated proton facility.

Taken together, while there is sound rationale, there is no strong clinical data to support the routine use of CPR for the treatment of HNSCC.

## 6. Conclusions

Randomized controlled trials have established IMRT as the current standard of care in the treatment of HNSCC. While technical advances in radiotherapy have revolutionized the treatment of HNSCC with the most tangible gain being a reduction in long-term morbidity, this success has not come without a price. Today, there is a greater chance of missing the target due to uncertainties in target volume definition by the clinician. Unless this is urgently addressed, we could be doing our patients a disservice as they would have been better off with a cure from HRT rather than IMRT that may be less morbid but does not cure them. We suggested potential solutions to circumvent this issue. IGRT ensures the level of accuracy warranted to deliver a highly conformal treatment plan and should be utilized with IMRT, where feasible. Guidelines for utilization of IGRT with IMRT have been provided. Proton therapy has a theoretical physical advantage over photon therapy. Although clinical data are currently sparse, from a historical perspective all techniques when initially introduced have suffered this disadvantage and are experimental/explorative to start with. Therefore, it is mandatory that every patient treated with this modality gets enrolled on a clinical study and data collated meticulously so we can document and learn from our experience and grow towards the future.
